# Genetic Architecture of Palm Oil Fatty Acid Composition in Cultivated Oil Palm (*Elaeis guineensis* Jacq.) Compared to Its Wild Relative *E. oleifera* (H.B.K) Cortés

**DOI:** 10.1371/journal.pone.0095412

**Published:** 2014-05-09

**Authors:** Carmenza Montoya, Benoit Cochard, Albert Flori, David Cros, Ricardo Lopes, Teresa Cuellar, Sandra Espeout, Indra Syaputra, Pierre Villeneuve, Michel Pina, Enrique Ritter, Thierry Leroy, Norbert Billotte

**Affiliations:** 1 Oil Palm Biology and Breeding Program, Corporación Centro de Investigación en Palma de Aceite (Cenipalma), Bogotá D.C., Colombia; 2 Umr Agap, Centre de coopération internationale en recherche agronomique pour le développement (CIRAD), Montpellier, France; 3 Laboratory of Molecular Biology, Empresa Brasileira de Pesquisa Agropecuária (EMBRAPA), Manaus, Brazil; 4 Agricultural Department, SOCFINDO (PT Socfin-Indonesia), Medan, Indonesia; 5 Umr Iate 1208, Centre de coopération internationale en recherche agronomique pour le développement (CIRAD), Montpellier, France; 6 Biotechnology Department, Instituto Vasco de Investigación y Desarrollo Agrario (NEIKER), Vitoria, Spain; Pennsylvania State University, United States of America

## Abstract

We searched for quantitative trait loci (QTL) associated with the palm oil fatty acid composition of mature fruits of the oil palm *E. guineensis* Jacq. in comparison with its wild relative *E. oleifera* (H.B.K) Cortés. The oil palm cross LM2T x DA10D between two heterozygous parents was considered in our experiment as an intraspecific representative of *E. guineensis*. Its QTLs were compared to QTLs published for the same traits in an interspecific *Elaeis* pseudo-backcross used as an indirect representative of *E. oleifera*. Few correlations were found in *E. guineensis* between pulp fatty acid proportions and yield traits, allowing for the rather independent selection of both types of traits. Sixteen QTLs affecting palm oil fatty acid proportions and iodine value were identified in oil palm. The phenotypic variation explained by the detected QTLs was low to medium in *E. guineensis*, ranging between 10% and 36%. The explained cumulative variation was 29% for palmitic acid C16:0 (one QTL), 68% for stearic acid C18:0 (two QTLs), 50% for oleic acid C18:1 (three QTLs), 25% for linoleic acid C18:2 (one QTL), and 40% (two QTLs) for the iodine value. Good marker co-linearity was observed between the intraspecific and interspecific Simple Sequence Repeat (SSR) linkage maps. Specific QTL regions for several traits were found in each mapping population. Our comparative QTL results in both *E. guineensis* and interspecific materials strongly suggest that, apart from two common QTL zones, there are two specific QTL regions with major effects, which might be one in *E. guineensis*, the other in *E. oleifera*, which are independent of each other and harbor QTLs for several traits, indicating either pleiotropic effects or linkage. Using QTL maps connected by highly transferable SSR markers, our study established a good basis to decipher in the future such hypothesis at the *Elaeis* genus level.

## Introduction

Indigenous to Africa, the oil palm (*Elaeis guineensis* Jacq.) is a perennial, monocotyledonous, monoecious, cross-pollinating species belonging to the *Arecaceae* family. The only other species in the genus *Elaeis* is the American oil palm, *Elaeis oleifera* (H.B.K) Cortés, indigenous to the Amazon region in South America [1], . Both species have 16 chromosome pairs (2n = 32) [3], and they can easily hybridize with each other [4].

Beginning the second year after planting and continuing throughout its life, the cultivated oil palm produces unisexual male or female inflorescences in successive cycles, emerging at the axil of each leaf. Female inflorescences grow in bunches that hold between 200 and 4 000 fruits. The oil palm fruit is a drupe. It comprises a pulp (mesocarp), an endocarp, called the *shell*; and a kernel. Three fruit types exist, depending on the presence or absence of the shell, which is governed by a major gene called *Sh* [5]. The *dura* type, homozygous *Sh^+^/Sh^+^*, produces large fruits with a thick shell and a pulp that is fairly abundant by weight (35–70%). The *pisifera* type, homozygous *Sh^−^/Sh^−^*, is generally female-sterile, and its few fruits are relatively small with a relatively large pulp (90%). The *tenera* type, heterozygous genotype *Sh^+^/Sh^−^*, thin shelled with an abundant pulp, produces the most palm oil and is therefore the fruit type of all commercial oil palm varieties. Only the thick-shelled *dura* type exists in the *E. oleifera* species.

The oil palm produces two distinct vegetal oils in its fruits: crude (red) palm oil, the fresh oil obtained from the mesocarp, and kernel oil, the oil obtained from the kernel seed of the palm fruit. These oils are used and marketed separately according to their own supply and demand conditions [6]. The oil productivity of the oil palm is ten-fold higher than that of the soybean, with a yield reaching 7.5 to 9 tons of oil/year/ha for the best Cirad commercial varieties in favorable agro-climatic conditions (www.palmelit.com). Palm oil constitutes the highest annual world production of vegetal oil, at 53.3 Mt, followed by soybean oil (43.4 Mt) and rapeseed oil (23.5 Mt) (http://www.fas.usda.gov/oilseeds/Current/). The wild species *E. oleifera* is not exploited at a commercial level due to its low yield [7].

The consumption of vegetal oils falls into two major applications: the food industry (with over 80% of the market) and the chemical industry [8]. Furthermore, in the 1980s, with the start of the full exploration of vegetable oils for biodiesel, principally due to interest in renewable energy sources, oil crops such as oil palm, soya, and oilseed rape began to be used as primary sources for these fuels, and higher proportions of unsaturated fatty acids are better for this purpose [9], .

Palm oil contains approximately 50% saturated fatty acids, with 44% palmitic acid (C16:0), 5% stearic acid (C18:0), and trace amounts of myristic acid (C14:0). The unsaturated fatty acids are approximately 40% oleic acid (C18:1) and 10% polyunsaturated linoleic acid (C18:2) and linolenic acid (C18:3) [11]–[13].

Currently, the need for high-oleic crops is increasing as the food market and the agro-industry demand oils that are more resistant to oxidation. Because of its saturated/unsaturated fat ratio close to 1 and its concentrations of carotenoids, tocopherols, and tocotrienols, palm oil is considered an oxidatively stable oil [6], [14] that has no equivalent among other vegetal oils. Another goal linked to the high oleic acid content is to improve the frying properties of palm oil for the food market, although the quality of palm oil in deep-fat frying methods has already been established [15]. Consequently, the improvement of palm oil quality for a higher degree of fatty acid unsaturation is one of the goals of oil palm breeders.

Several studies have investigated the phenotypic characterization and genetic determinants of the palm oil fatty acid composition [12], [16]–[18]. These studies showed that there is significant variability in the fatty acid composition among *E. guineensis* populations. In addition, the composition of palm oil is substantially different between *E. guineensis* and *E. oleifera*, the latter characterized by its higher content of unsaturated fatty acids. Among the *E. oleifera* palms, the unsaturated fatty acid content ranges from 47% to 69% for C18:1, 2% to 19% for C18:2, and 0.1% to 1.2% for C18:3 [19]–[21]. Consequently, the iodine value (IV), a multi-parameter measure of the global degree of unsaturation of the fatty acids in vegetal oil, is higher in *E. oleifera* than in *E. guineensis* due to the major oleic acid C18:1 and the linoleic acid C18:2. The IV for *E. oleifera* is between 70% and 87% [13], [19], [20], [22], whereas the value for *E. guineensis* varieties is between 53% and 60% [12], [22]–[24]. Studies in interspecific hybrids showed that the palm oil composition profile is intermediate between those of the two parental *Elaeis* species for most fatty acids, which is an advantage for introgressing these traits from *E. oleifera* into varieties of *E. guineensis* [4], [7], [25]. As far as we know, only two QTL studies have been performed for palm oil fatty acid composition, one in oil palm [60], the other one in an interspecific pseudo-hybrid [26], and no published study has compared QTLs of the palm oil fatty acid composition between the two *Elaeis* species or between intraspecific and interspecific materials except for Montoya *et al.* [27].

According to the review by Murphy [28] and references therein, the oil crop varieties that have acquired significantly increased unsaturated fatty acid proportions by conventional breeding methods are the rapeseed/canola, soybean, sunflower and safflower (with 75% oleic acid and 1% to 3% linolenic acid), and the olive (with 85% oleic and 1% linolenic acid). Additionally, transgenic approaches have produced varieties that have significantly increased unsaturated fatty acid content: rapeseed/canola, with 89% oleic acid; Indian mustard, with 73% oleic acid; soybean, with 75% oleic acid; and cotton seed, with 78% oleic acid. One breeding strategy to achieve a highly unsaturated palm oil is to take advantage of the unsaturated profile found in *E. oleifera* using interspecific hybridization or backcross strategies [4], [7], [29].

Few authors have reported data describing correlations, at the level of individual palm trees between fatty acid proportions in the *Elaeis* genus. Even fewer data have been published regarding the correlation of fatty acid proportions with vegetative and production traits in support of breeding strategies to modify the palm oil composition.

By contrast, in this respect, there is evidence of interference between agronomic traits and oil quality, as reported for the soybean. Several studies have shown that the presence of major and minor genes that reduce the palmitate (C16:0) content in seed oil also reduces seed yield or plant height due to pleiotropic effects or linkage with unfavorable yield genes [30], [31]. However, the increased oleic acid in soybeans with high-yielding genetic backgrounds does not affect yield or other agronomic traits [32], [33].

In the olive tree (*Olea europaea* L.), correlations between fruit characteristics, oil yield components, and fatty acid composition in progenies from different crosses show significant positive relationships between oil content and oleic acid percentage, which are negatively correlated with the palmitic, palmitoleic, and linoleic acid contents [34].

The principal aim of the present study was to identify the genetic determinants of the palm oil fatty acid composition in the cultivated oil palm in comparison with its wild relative *E. oleifera*. As a preliminary study, the palm oil composition was characterized in an intraspecific *E. guineensis* cross, and the relationships between its components were studied to better understand the genetic determinants of palm oil fatty acid proportions. In addition, relationships with other production traits were studied to determine whether selection for a modified palm oil composition could significantly influence the palm oil yield traits. Based on the phenotypic variability existing between *E. guineensis* and *E. oleifera* in the profile of fatty acids of their respective oils, we hypothesized that the genetic architecture of this trait would differ between these *E. guineensis* intra-specific and interspecific mapping populations, in terms of the number and position of QTLs and in the phenotypic variance they explain. To test this hypothesis, two *Elaeis* mapping populations were exploited: an *E. guineensis* intraspecific cross and an interspecific pseudo-backcross. A second hypothesis was that the two *Elaeis* genomes would have good locus collinearity because they are not distant relatives, as they intercross easily. In these crosses, dense microsatellite linkage maps, with a high number of common simple sequence repeat (SSR) marker loci, allowed us to identify and compare QTLs. The results of the QTL detection undertaken in the *E. guineensis* cross were interpreted in comparison with QTLs for the same traits in the interspecific pseudo-backcross.

Previous QTL detections for palm oil fatty acid composition in the *Elaeis* genus [26], [60] where unfortunately based on incomplete linkage maps mostly using AFLP, RFLP, or SSCP non-transferable marker loci. Such situations did not allow to relate and to compare QTL results, as well as with ours and those of Montoya *et al.* [27] which are in the contrary based on a saturated linkage map, made of SSR marker loci higly polymorphic and transferable within the *Elaeis* species. Therefore, we chose to use same linkage map quality and related *E. guineensis* map parents in our comparative experiment.

## Materials and Methods

### Vegetal material

The *E. guineensis* cross LM2T x DA10D, previously described by Billotte *et al.* [35], was used to perform a QTL analysis of fatty acid proportions in palm oil from mature fruits. The progeny consisted of 116 full-sibs derived from the cross between two heterozygous *E. guineensis* parents from the CNRA oil palm breeding program (La Mé, Côte d'Ivoire): LM2T, a *tenera* palm belonging to the La Mé African population, and DA10D, a *dura* palm selected from the Deli population. In this cross, traceable DNA marker alleles (and detectable QTL marker alleles) are those segregating from heterozygous loci of one, the other or both *E. guineensis* map parents (Figure S1A).

We compared the intraspecific QTLs identified in LM2T x DA10D to those shown to be responsible for the same traits in an *Elaeis* interspecific pseudo-backcross, SA569, described by Montoya *et al.* [27]. The SA569 mapping population consisted of 134 full-sibs derived from an interspecific palm, SA65T (*E. oleifera* SA49D x *E. guineensis* LM2466P), and an *E. guineensis* genitor, PO3228D. The female grandparent (SA49D) was a wild *E. oleifera* palm (*dura*) from the Coari region (Brazilian Amazon), and the male grandparent (LM2466P) was a *pisifera* oil palm obtained by selfing the LM2T oil palm genitor (parent of the intraspecific *E. guineensis* cross mentioned above). The *E. guineensis* male parent PO3228D was a *dura* oil palm derived by selfing the DA115D oil palm genitor of the Deli population. Sharing same Deli ancestors, DA115D (subsequently its self PO3228D) is genetically very close to the parent DA10D of the *E. guineensis* cross mentioned above. The *E. guineensis* grand-parent (LM2466P) and parent (PO3228D) are highly homozogous, over 75% [27]. By genetic construction of the pseudo-backcross, most traceable DNA marker loci are segregating only from heterozygous loci in the interspecific parent SA65T, each of them holding one *E. guineensis* allele (from LM2T or DA115D closely related to DA0D) and one *E. oleifera* allele (Figure S1B). Most QTL would be polymorphic and detectable on the interspecific parent SA65T only, while most of these QTL will be monomorphic and not identifiable on the *E. guineensis* parent PO3228D, but detectable in the LM2T x DA10D cross when existing and heterozygous on the *E. guineensis* LM2T or DA10D parent.

### *Elaeis* linkage map system for intra- and interspecific QTL comparison

#### 1) Intraspecific multi-parent SSR consensus map in *E. guineensis*

This map, published by Billotte et al. [36] and henceforth referred to as Eg_Map, was constructed using SSRs genotyped in a 2×2 complete factorial mating experiment of four unrelated parents belonging to the La Mé population from Africa (tenera LM2T), the Yangambi population from Africa (tenera LM718T), and the Deli population (dura DA10D, dura LM269D). Each family was a single cross between two heterozygous parents, including one tenera parent from Africa and one dura parent of the Deli population: LM2T x DA10D (mentioned above), LM2T x LM269D, LM718T x DA10D, or LM718T x LM269D.

These four crosses used by Billotte *et al.* [36] to establish the consensus Eg_Map were part of a larger genetic trial begun in 1986 by the company SOCFINDO (Medan, Indonesia), whose experimental design was a randomized complete block design (RCBD) with five replications of 15 palms. The agro-climatic conditions were highly favorable for oil palm growth. The 116 LM2T x DA10D full-sibs described above were part of that experiment, and all other crosses were represented by 61 palms (a total of 299 palms for Eg_Map). The consensus Eg_Map, including 16 linkage groups, had 253 loci (251 SSRs, the *Sh* locus, which controls shell thickness, and its marker E-Agg/M-CAA132) and measured 1 731 cM (Haldane distance), with an average marker density of 7 cM [36].

#### 2) *Elaeis* interspecific linkage map

The second genetic map used in this study was a dense SSR-based linkage map made from the interspecific pseudo-backcross SA569 described above, as published by Montoya *et al.* [27]. In this pseudo-backcross, molecular marker alleles were traced that segregated from both *E. oleifera* and *E. guineensis* grandparents and then from the interspecific and *E. guineensis* parents. The SSR map of SA569 had 362 loci (347 SSRs, 14 SNPs, and the *Sh* locus) and measured 1 485 cM (Haldane distance), with an average marker density of 4 cM. In total, 156 marker loci (155 SSRs + *Sh*) were shared in common by *E. oleifera* and *E. guineensis*, and there was good marker co-linearity with Eg_Map, enabling the comparison of intra- and interspecific QTLs for fatty acid composition.

### Measurements of the palm oil fatty acid composition in LM2T x DA10D

The LM2T x DA10D progenies, as well as five LM2T self palms and five DA10D self palms, were analyzed for fatty acid composition and for the iodine value (IV) of the palm oil in mature fruits. The trait mean value from the LM2T and DA10D self palms gave an estimate of the LM2T and DA10D parent trait values. Of the 116 progenies planted initially for this cross, only 88 were available that had produced bunches and allowed for estimates of the posterior palm oil fatty acid composition and iodine value due to the death of some palms before our phenotypic characterization in 2012.

Measurements of the palm oil fatty acid composition in the LM2T x DA10D cross were performed as per Montoya *et al.* [27]. Quantitative phenotypic traits in this study considered the nine main fatty acids: myristic acid (C14:0), palmitic acid (C16:0), palmitoleic acid (C16:1), stearic acid (C18:0), oleic acid (C18:1), linoleic acid (C18:2), linolenic acid (C18:3), arachidic acid (C20:0), and gadoleic acid (C20:1). The iodine value (IV) was determined by the Wijs method described in the ISO 3961:2009 standard. For the IV, mean values were estimated for all 116 palms (based either on four measurements recorded in 2002–2004 and 2012 for the 88 surviving palms or on two previous measurements in 2002–2004 for palms that had died since 2004).

Regarding the interspecific pseudo-backcross SA569, a total of 115 progeny, as well as the *E. oleifera* grandparent SA49D, the interspecific parent SA65T, and the *E. guineensis* parent PO3228D, had been previously analyzed by Montoya *et al.* [27] for fatty acid composition and the iodine value of the palm oil in mature fruits. Available individual phenotypic data were used here for a comparative study with the LM2T x DA10D cross.

### Other phenotypic traits and QTL data available for LM2T x DA10D and SA569

The fruit variety and the individual phenotypic data of 26 vegetative or yield quantitative traits were available from Billotte *et al.* [36] for the four crosses of Eg_Map, including LM2T x DA10D (Table S1). The main quantitative variables of production that we considered were the average bunch number/palm/year at 3–5 years (Bn3_5), average bunch weight at 3–5 years (kg) (Bwt3_5), average bunch number/palm/year at 6–9 years (Bn6_9), average bunch weight at 6–9 years (kg) (Bwt6_9), average number of spikelets per bunch (Spikelets), average number of fruits per bunch (Fn), fruit to bunch ratio (%FB), average fruit weight (g) (Fwt), pulp to fruit ratio (%PF), palm oil to pulp ratio (%POP), kernel to fruit ratio (%KF), and iodine value (IV). Except the fruit type, no vegetative or production traits were available for the pseudo-backcross SA569.

The QTL information published by Billotte *et al.* [36] for the 26 vegetative and production traits and for the iodine value (IV) associated with the palm oil fatty acid profile in *E. guineensis* (QTL position, confidence interval) were available for Eg_Map and therefore could be compared to the QTL information for palm oil fatty acid composition identified for LM2T x DA10D using Eg_Map. Based on the SA569 map, 19 QTLs for palm oil fatty acid composition and 14 *Elaeis* intra-gene SNP markers for five gene functions associated with oleic acid C18:1 in palm oil were available from Montoya *et al.* [27].

### Statistical analysis of phenotypic data

The iodine value was calculated from the average of all measurements made per palm, as described above. Statistical analyses were performed on phenotypic data for fatty acid composition in the LM2T x DA10D cross. The Gauss distribution of the quantitative data of the cross was checked by the Shapiro-Wilk normality test at the *α* threshold of 5%. The relationships between phenotypic traits published by Billotte *et al.* [36] and the fatty acid proportions estimated in the LM2T x DA10D cross were estimated by calculating Pearson's correlation coefficients at the individual palm level (Table S2).

In a second step, the vegetative and production phenotypic data of LM2T x DA10D were standardized as the mean and variance for both *dura* and *tenera* varieties to eliminate the *Sh* major gene effect on traits. The fatty acid composition data were not standardized because very few, negligible correlations existed with the fruit type. All these data were subjected to an overall Pearson's correlation analysis to determine, at the individual palm level, the relationships between the fatty acid proportions and the vegetative and production traits.

Principal component analysis (PCA) provides a synthetic vision of the relationships between studied variables based on estimated correlations. Every principal component (PC), a linear combination of variables, is mathematically orthogonal to every other PC. Different PCs illustrate the degree of independence between their respective contributing variables. PCA was carried out using XLSTAT statistical software (Addinsoft, NY, USA) to visualize the associations among yield and palm oil composition traits, at the *E. guineensis* intraspecific level, using the Pearson's correlation matrix of LM2T x DA10D.

Associations among palm oil fatty acid composition traits were analyzed at both the intraspecific and interspecific levels by performing a PCA of five major fatty acids (C14:0 C16:0, C18:0, C18:1, and C18:2) in LM2T x DA10D and in SA569.

### QTL analysis of LM2T x DA10D

QTL analyses of palm oil fatty acid composition were performed with MapQTL5 [37] using the *E. guineensis* SSR linkage map (Eg_Map) and all available genotypic and phenotypic data for LM2T x DA10D. No QTL analysis of other vegetative or production traits was performed here, as related QTLs are already available in Billotte *et al.* [36].

Three methods were used for QTL detection as per Montoya *et al.* [27]: i) A non- parametric Kruskal-Wallis (K-W) test was performed to identify significant marker-trait associations at p<0.005. ii) The interval mapping (IM) method was performed with a mapping step size of 1 cM and a maximum of five neighboring markers. To declare the presence of a QTL, the threshold LOD values were estimated at the genome-wide (GW) global risk α of 5% and 1% by the re-sampling method and permutation of the trait data (1000 iterations). iii) The multiple-QTL model (MQM) method was carried out in conjunction with the automatic selection of cofactors, using the threshold LOD values described above. The threshold value of p<0.005 in the non-parametric K-W method was adopted on a empiric basis, as it was equivalent in QTL results compared to a GW global risk α of 5% using the different IM and MQM methods. A GW global risk α of 1% was also used in the IM or MQM method to explore the robustness of detected QTL.

The confidence interval of each significant QTL by IM or MQM was determined by the LOD –1 method. For the phenotypic values of fatty acids (C16:1, C18:3, C20:0 and C20:1) in trace or in small amounts that did not follow a mixture normal distribution (Figure S2), the Kruskal-Wallis rank sum test was only considered as applicable for data with distributions far from mixture normal distribution [38]. A limited population size for identifying QTLs affects the accuracy of determining QTL locations and estimating QTL effects and, consequently, overestimates the phenotypic variances associated with QTLs [39]–[41]. To correct at least the small part of the overestimation due to sampling error, we applied the correction described in Montoya et al. [27], as proposed by Luo et al. [42] and Xu [43]. They suggest to multiply the explained variance by 1−1/(2*Ln(10)*LOD).

Therefore, considering that the variance explained by an identified QTL is, as estimated under MapQTL,



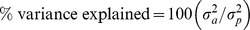
with σ_a_^2^ corresponding to the genetic variance due to additive effect and σ_p_^2^ corresponding to the phenotypic variance,

the corrected variance explained by this QTL was re-estimated like follows:
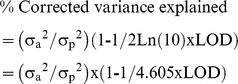
where LOD corresponds to the LOD value of the identified QTL.

## Results

### Palm oil fatty acid composition in the *E. guineensis* cross LM2T x DA10D

The results for palm oil fatty acid proportions found in the LM2T x DA10D cross are given in Table 1 and Table S1. The principal fatty acids were palmitic acid (C16:0, mean 40.5%) and oleic acid (C18:1, mean 43.5%), followed by linoleic acid (C18:2, mean 9.3%) and stearic acid (C18:0, mean 5.2%). The higher coefficients of variation were for the fatty acids present in trace amounts (means <1%), such as C14:0, C16:1, C18:3, C20:0, and C20:1. The ratio of saturated (46.6%) to unsaturated (53.4%) fatty acids was in accordance with the 1:1 ratio, as demonstrated by a χ^2^ test (data not shown).

**Table 1:** Means, ranges, variances, and coefficients of variation (CVs) for palm oil fatty acid composition and iodine value in the *E. guineensis* intraspecific cross LM2T x DA10D.

Traits	Mean (n = 88)	Range	Variance	CV a (%)	LM2T self Mean (n = 5)	DA10D self Mean (n = 5)
C14:0	0.5	0.3–1.0	≈0.0	32.0	0.2	1.3
C16:0	40.5	32.5–50.0	9.2	7.5	32.9	45.9
C16:1	0.1	0.1–0.2	≈0.0	30.2	0.1	0.1
C18:0	5.2	3.7–8.4	0.8	17.1	6.5	4.1
C18:1	43.5	35.3–50.0	7.6	6.3	49.9	35.4
C18:2	9.3	5.0–12.2	1.2	11.5	9.0	12.3
C18:3	0.2	0.1–0.4	≈0.0	21.7	0.3	0.3
C20:0	0.3	0.2–0.5	≈0.0	19.3	0.3	0.3
C20:1	0.1	0.1–0.3	≈0.0	37.0	0.1	0.1
Saturated	46.6	41.0–54.4	6.4	5.4	39.9	51.7
Monounsaturated	43.6	27.2–50.0	10.6	7.5	50.1	35.6
Polyunsaturated	9.8	7.1–26.0	4.0	20.4	9.3	12.6
Iodine value	55.3	49.4–61.2	4.0	3.6	59.4	52.7

a CV: Coefficient of variation.

The mean fatty acid contents of the progeny were equal to the mean values estimated for their parents LM2T and DA10D, except for C18:2, as demonstrated by a χ^2^ test (data not shown).

The normality test (data not shown) showed a normal distribution for C16:0, C18:1, and IV but not for the other fatty acids. The histograms (Figure S2) showed discontinuous variations for C16:1, C18:3, C20:0, and C20:1, and for this reason, they were not considered for further analysis of QTLs.

In the PCA for the LM2T x DA10D cross with 16 elementary variables associated with agronomic traits and palm oil fatty acid composition (data not shown), the first four components explained 61% of the global phenotypic variation, indicating the existence of four groups of correlated traits. The variation explained by each component was PC1 21.9%, PC2 38.6%, PC3 51.3%, and PC4 61.0%. The PC1 had loadings mainly for fatty acid traits (C14:0, C16:0, C18:0, and C18:1), whereas the PC2 was for traits associated with oil palm production (Bwt3_5, Bn6_9, and Bwt6_9, Spikelets), the PC3 was for fruit pulp-related traits (%PF, %POP, and %KF), and the PC4 was for fruit production traits (Fn, Fwt, and %FB).

The Pearson's correlation coefficients (Table 2) for the most relevant fatty acids showed that C14:0 was positively correlated with C16:0 and C18:3 and negatively correlated with C18:0, C18:1, and IV. Palmitic acid C16:0 was negatively correlated with C18:0, C18:1, and IV. Stearic acid C18:0 was positively correlated with C18:1 and IV. Oleic acid C18:1 was negatively correlated with C18:2 and C18:3 and positively correlated with IV. Considering all vegetative, production, and fatty acid composition traits, few correlations were significant at p<0.05 between a fatty acid proportion and a vegetative or production trait (Table S2). Globally, fatty acid proportions were not correlated with the vegetative or production traits under study, i.e. they were statistically independent, except for the bunch number and the number of leaflets per mature leaf of rank 17.

**Table 2:** Individual Pearson's correlation coefficients for fatty acid proportions and iodine value (IV) in the *E. guineensis* intraspecific cross LM2T x DA10D.

Traits	C16:0	C16:1	C18:0	C18:1	C18:2	C18:3	C20:0	C20:1	IV
C14:0	0.73**	0.39**	−0.60**	−0.73**	0.13	0.38**	−0.40**	−0.11	−0.64**
C16:0		0.37**	−0.77**	−0.92**	0.06	0.21	−0.43**	−0.14	−0.86**
C16:1			−0.38**	−0.38**	0.13	0.34**	−0.45**	−0.02	−0.25*
C18:0				0.60**	−0.04	−0.15	0.50**	0.09	0.55**
C18:1					−0.39**	−0.25**	0.34**	0.13	0.74**
C18:2						0.09	−0.01	−0.12	0.22
C18:3							−0.15	−0.07	−0.12
C20:0								0.12	0.38**
C20:1									0.11

Asterisks indicate significant correlations at *: p≤0.05 or **: p≤0.01

### Relationships between the five main fatty acid proportions in *E. guineensis* and *E. oleifera*

The results of the two PCAs for only palm oil fatty acid composition in LM2T x DA10D and SA569 showed that the first three components explained 93.2% and 87.3% of the global variation in LM2T x DA10D and SA569, respectively, representing high percentages of same order of magnitude (Table 3). The factor loadings of the linoleic acid C18:2 showed that the latter was independent of the other main fatty acid traits in the intraspecific or interspecific genetic material. However, different tendencies were found in the correlations between the five main fatty acid proportions, depending on the genetic material. Thus, in the *E. guineensis* cross, the fatty acid proportions of C14:0, C16:0, C18:0, and C18:1 were all highly correlated with each other (as mainly represented by PC1), whereas in the interspecific SA569, C16:0 and C18:1 (associated with PC1) were correlated with each other but were independent of C14:0 and C18:0 (associated with PC2), which, in turn, were correlated with each other.

**Table 3:** Principal component analysis of five main fatty acid composition traits in both intraspecific cross LM2T x DA10D and interspecific pseudo-backcross SA569.

Item	Acronym	LM2TxDA10D			SA569		
		PC1	PC2	PC3	PC1	PC2	PC3
Variation explained (%)		63.2	21.3	8.7	41.1	27.8	18.4
Accumulated variation explained (%)		63.2	84.5	93.2	41.1	68.9	87.3
Myristic acid (%)	C14:0	**0.85**	−0.02	0.29	−0.43	**−0.64**	0.23
Palmitic acid (%)	C16:0	**0.95**	−0.19	0.03	**−0.81**	−0.32	−0.43
Stearic acid (%)	C18:0	**−0.80**	0.25	0.53	−0.15	**0.83**	−0.30
Oleic acid (%)	C18:1	**−0.92**	−0.19	−0.20	**0.97**	−0.23	0.10
Linoleic acid (%)	C18:2	0.19	**0.96**	−0.17	−0.50	0.38	**0.76**

The variation explained and the factor loadings for the three first principal components are shown. The values for traits with the highest factor loadings for a principal component are shown in bold.

**Figure 1:**
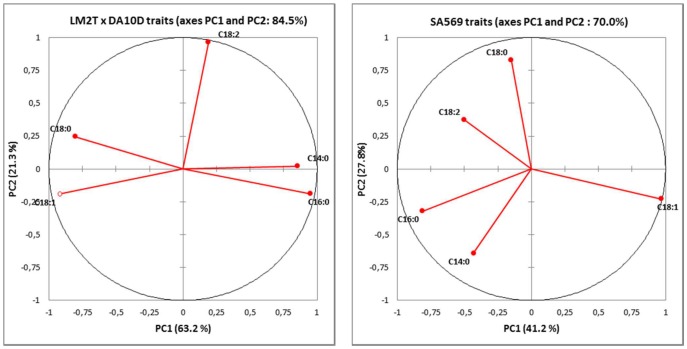
Principal component analysis (PCA) of palm oil fatty acid proportions of C14:0, C16:0, C18:0, C18:1 and C18:2 in the intraspecific cross LM2T x DA10D and in the interspecific pseudo-backcross SA569. Note: the figure show projections on the two first axes of the PCA.

The projection of the variables in the PC1-PC2 plans illustrates these principle relationships, as shown in Figure 1.

### QTLs involved in fatty acid composition

**Figure 2:**
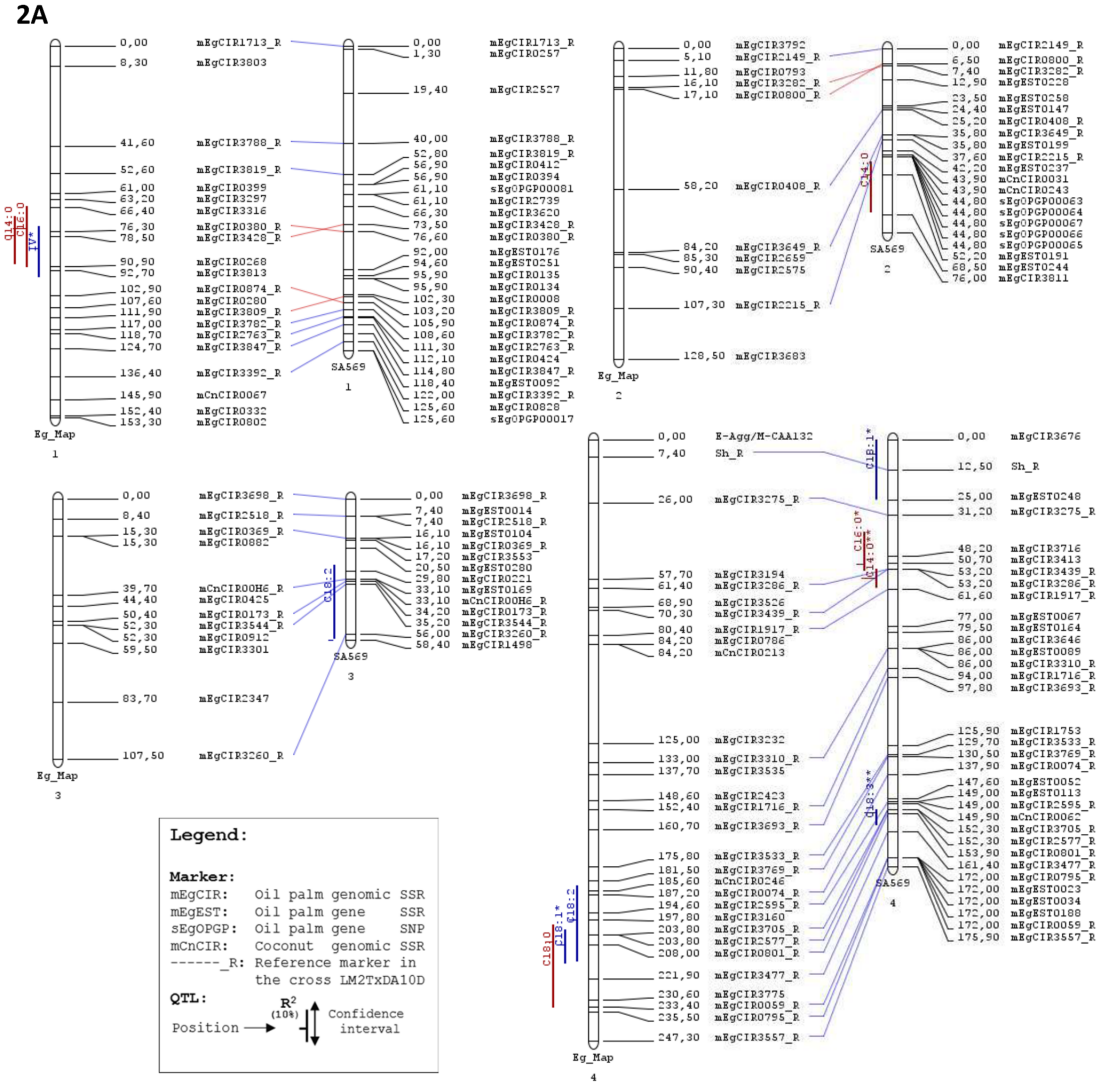
Sixteen QTLs of palm oil fatty acid proportions and iodine value identified in the *E. guineensis* cross LM2T x DA10D, located on the consensus linkage map in oil palm (Eg_Map) of Billotte *et al.* [36] and compared to the QTL map for same traits published by Montoya *et al.* [27] in the interspecific *Elaeis* pseudo-backcross SA569. Note: Each microsatellite linkage map has 16 linkage groups corresponding to the 16 homologous pairs of chromosomes of the *Elaeis* genome. The *E. guineensis* Eg_Map (253 loci) is sharing 156 marker loci in common and good co-linearity with the linkage map of the pseudo-backcross SA569 (362 loci). The QTLs were identified by the Kruskal-Wallis, IM and MQM methods. One star (*) or two stars (**): QTL detected by the MQM method at the genome-wide α threshold value of 5% or 1% respectively. No star: putative QTL as only detected by the Kruskall-Wallis test at p<0.005. The names and the positions (cM) of the markers are given on the right side of the linkage groups. mEgCIRxxxx and mEgESTxxxx: *E. guineensis* SSR loci. sEgOPGPxxxx: *E. guineensis* gene SNP loci. mCnCIRxxxx: *Cocos nucifera* SSR loci. Marker loci common to both maps are indicated by an extension “_R”. The names, positions and confidence regions of the QTLs are given on the left side of the linkage groups. In red: are figured the QTLs of saturated fatty acid proportion; in blue: the QTLs of unsaturated fatty acid proportion and of iodine value.

Sixteen QTLs associated with palm oil fatty acid composition were evidenced by the Kruskal-Wallis (K-W) analysis, with one to three QTLs per fatty acid or iodine value (IV) (Table 4 and Figure 2). QTLs only detected by the K-W method and considered “putative” were mapped in linkage groups (LGs) 1, 4, and 13. For these putative QTLs, a peak LOD value was observed with the IM and/or MQM methods at the same or a nearby location, although not significant. Nine QTLs were confirmed by IM and ten QTLs by MQM, at the significant genome-wide threshold α of 1% or 5%. These latter QTLs were located in five LGs (4, 8, 9, 14, and 15) of Eg_Map.

**Table 4:** List of QTLs identified by the Kruskal-Wallis method (at p<0.005) and by the interval mapping (IM) and/or multiple-QTL Model (MQM) methods for fatty acid composition in the cross LM2Tx DA10D.

Trait	Interval Mapping analysis								MQM analysis					
	LG^a^	QTL peak (cM)^b^	Marker c	Maximum LOD^d^	MapQTL estimated Expl. Var e	LG^a^	QTL peak (cM)^b^	Marker c	Maximum LOD^d^	% Expl.^e^	Corrected Expl. Var. e	GW^f^		Confidence Interval (cM)
C14:0	1	78.5	mEgCIR3428	3.1	19.4							5.0	6.1	70.4–90.5
C14:0	9	9.8	mEgCIR3787^$^	6.2**	31.6	9	9.8	mEgCIR3787^$δ^	6.3**	31.6	30.5			0.0–25.6
C16:0	1	75.4	mEgCIR0308^$^	2.4	26.0									66.2–91.6
C16:0	9	0.0	mEgCIR2224	6.0**	30.3	9	0.0	mEgCIR2224^δ^	6.0**	30.3	29.2	4.3	4.9	0.0–7.0
C16:1	*not analysed*													
C18:0	4	206.8	mEgCIR0801^$^	2.6	13.9									199.8–234.4
C18:0	9	0.0	mEgCIR2224	6.0**	33.4	9	0.0	mEgCIR2224^δ^	6.0**	33.4	32.2	4.6	5.6	0.0–5.8
C18:0	13	51.2	mCnCIR0038	2.4	11.7									44.5–84.4
C18:0	-	-	-	-	-	14	50.1	mEgCIR3546	4.7*	38.0	36.2			40.1–65.7
C18:1	4	208.0	mEgCIR0801	4.3*	20.1	4	208.0	mEgCIR3160	4.4*	10.3	9.8	4.3	5.0	201.8–216.0
	9	0.0	mEgCIR2224	6.6**	36.4	9	0.0	mEgCIR2224^δ^	6.7**	27.6	26.7			0.0–6.0
	15	30.1	mEgCIR0409	4.4*	23.1	15	30.1	mEgCIR0409^δ^	4.4*	14.4	13.7			25.6–34.9
C18:2	4	197.8	mEgCIR3160	3.0	15.4									183.5–215.0
C18:2	8	186.0	mEgCIR2887	5.7**	25.7	8	186.0	mEgCIR2887^δ^	5.7**	25.7	24.7	4.2	5.0	174.8–189.1
C18:3	*not analysed*													
C20:0	*not analysed*													
C20:1	*not analysed*													
IV^g^	1	83.5	mEgCIR3428^$^	4.4*	19.5	1	83.5	mEgCIR3428^$δ^	4.5*	16.6	15.8	4.1	4.8	74.4–95.7
	15	29.3	mEgCIR3346	7.4**	28.2	15	29.3	mEgCIR3428^$δ^	7.2**	25.4	24.6			26.9–32.7

a LG  =  Linkage group

b Cummulative distance from the top marker of the linkage group

c $: Neighborhood locus if not at the QTL position; δ: Cofactor marker for MQM analysis

d α significance threshold: ** at 5%. * at 1%

e Percentage of the phenotypic variance explained at the QTL

f α genome-wide significance threshold at 5% or 1% of level of probability

g IV: Average value estimated from two repetitions per palm in 2002–2004 and 2012.

The percentage of the phenotypic variation explained by a significant QTL corrected for the sampling error was low to medium and ranged between 10% and 36% (Table 4). The total phenotypic variation explained by the QTLs for the principal fatty acids was 29% for C16:0 (one QTL), 68% for C18:0 (two QTLs), 50% for C18:1 (three QTLs), and 25% for C18:2 (one QTL). The explained cumulative variation for IV was 40% (two QTLs). We did not estimate the LM2T or DA10D parent effect at QTLs, as such an estimation would require a larger mapping population of at least 200 palms.

Several QTLs were closely linked or co-localized. LG 9 showed a co-localization of three QTLs for the traits C16:0, C18:0, and C18:1 at the position 0.0 cM and a neighborhood QTL for C14:0 at 9.8 cM. Putative QTLs in LG 4 for C18:0 and C18:2 were closely linked along with a QTL for C18:1 determined by IM and MQM. LG 1 contained closely linked putative QTLs for C14:0 and C16:0 and another QTL for IV. Other QTLs for C18:1 and IV were closely linked in LG 15. Finally, LG 14 and LG 13 presented one QTL for C18:0 and LG 8 one QTL for C18:2.

In general, no position correspondence was found between intraspecific QTL regions identified on Eg_Map and interspecific QTL regions identified previously by Montoya *et al.* [27] in the pseudo-backcross SA569. The two genetic materials showed only two cases with QTLs in the same regions: at the bottom of LG 4 (around mEgCIR0801) and at the top of LG 15, showing C18:1 and IV QTLs in Eg_Map and C16:0 and IV putative QTLs in SA569.

There were two main independent QTL regions, one in the *E. guineensis* cross (in LG 9) and the other in the *E. oleifera*-derived cross (in LG 6), with major effects harboring QTLs for several traits with high effects.

## Discussion

### Palm oil fatty acid composition in LM2T x DA10D

Our phenotypic data for fatty acid composition show wide variation within the cross, with low individual values for palmitic acid C16:0 (32.5%) and high values for oleic acid C18:1 (50%). The mean 1∶1 ratio of saturated to unsaturated fatty acids was in accordance with Ebong *et al.* [14]). Our data are consistent with other reports for genetic materials descending from the La Mé origin, characterized by its relatively low amount of palmitic acid and high amount of oleic acid in comparison to other *E. guineensis* origins [44]. La Mé x Deli crosses have concentrations of 40% and 41% for palmitic acid and oleic acid, respectively [11], [16]. More recently, Monde *et al.* [45] evaluated La Mé and Deli collections from Côte d'Ivoire. These authors recorded concentrations of 31% for palmitic acid and 50% for oleic acid from La Mé, whereas from Deli, they recorded concentrations of 45% and 38% for palmitic acid and oleic acid, respectively.

The above findings imply that the breeding populations La Mé and Deli are two sources of variability in fatty acid composition and consequently provide segregating progenies for evaluating genetic variation and searching for QTLs related to the fatty acid composition of *E. guineensis* mature fruits. At the same time, the La Mé and Deli origins, which are among the major populations used by oil palm breeders, represent an important genome resource for identifying allelic variants within the *E. guineensis* species of genes involved in palm oil biosynthesis and in determining the final proportion of fatty acids in mature fruits. Our LM2T x DA10D is a valuable reference and starting point in that respect.

[7] and [25] have reported that most fatty acid proportions and total unsaturated fats in *Elaeis* interspecific hybrids are intermediate between the parents' proportions, indicating these are quantitative traits with additive effects, with the exception of linoleic acid (C18:2), for which *E. guineensis* seemed to be dominant for its corresponding allelic genetic factors. Our study clearly confirmed the additive genetic determinants of fatty acid proportions in our *E. guineensis* cross, as the mean values of LM2T x DA10D progeny were the mean values of their parents, except for C18:2, which showed a non-additive genetic determinism, with LM2T appearing dominant over DA10D, consistent with Hardon [7].

Similarly, our results on the interspecific pseudo-backcross are consistent with Tan *et al.* [23], who reported a co-dominant heredity in hybrid progenies, with the same exception of linolenic acid (C18:2), whose effects seem to be dominant in *E. guineensis*. Our PCA based on the fatty acid variables showed a relative independence of linoleic acid C18:2 compared to other fatty acids. This independence might correspond to the *de novo* fatty acid synthesis that occurs in the plastid (C14:0, C16:0, C18:0, and C18:1), under the control of genes with additive effects, whereas C18:2, a fatty acid whose elongation and desaturation occur in the endoplasmic reticulum, might be under the main control of a gene(s) with dominant effects.

Our phenotypic data and those of Montoya *et al.* [27] showed intra- and interspecific variability and mainly additive genetic determinism for *Elaeis* palm oil composition. This finding implies the strong possibility of using genetic manipulation to improve the unsaturated fatty acid proportions of palm oil based on the genetic values of the genitors to be selected.

### Correlations with production traits

Our estimated correlations between fatty acid proportions in the pulp of mature fruits were similar to those of Noiret and Wuidart [17] and Wuidart and Gascon [16] in La Mé x Deli crosses and to those of Noh *et al.* [12] in the *E. guineensis* germplasm collected from Angola. There were also similarities in the interspecific pseudo-backcross SA569 involving *E. oleifera* [27] and in a pseudo-hybrid [26]. This agreement in findings tends to confirm that such individual correlations are valid for the whole *Elaeis* genus.

An important new finding of this study is a general correlation table between vegetative, production and palm oil fatty acid traits, which was never published in oil palm. That latter showed few correlations between the palm oil fatty acid composition and the elementary production traits in the *E. guineensis* cross, such like between the bunch number at the young age with C16:0, C18:0 and C18:1. We remark as well similar correlations between these fatty acid proportions and the number of leaflets per leave at the adult age. As far as we know, no individual phenotypic correlations have been reported between palm oil fatty acid proportions and vegetative or production traits of oil palm. Correlations exist between fatty acid composition and production in other species, such as the olive tree [34], [46] and *Brassica napus* [47]. Our results indicate that the palm oil fatty acid proportions of mature fruits are globally not correlated with elementary vegetative or production traits, while few correlations with some production traits should not be ignored. This finding suggests that breeding to modify the palm oil composition can be performed in *E. guineensis* without affecting important bunch components, such as the percentage of pulp on the fruit and the oil content of the pulp, which are key parameters in the elaboration of the final palm oil yield. Meanwhile, some cautions should be taken regarding the bunch number. According to Billotte *et al.* [36], who studied 1,182 palms from 16 different full-sib families, the iodine value was also positively correlated (p<0.01) with the percentage of palm oil in the pulp (%POP). Here, that correlation was not significant in 88 LM2T x DA10D individuals, although our samples had a narrower genetic base. Interpretation at the level of the *Elaeis* genus must be supported by further experiments using a large panel of various *E. guineensis*, *E. oleifera*, and interspecific populations.

### Genetic information from mapped QTLs and breeding perspectives for palm oil composition

LM2T x DA10D was considered as a representative of pure intraspecific *E. guineensis* materials and the interspecific pseudo-backcross SA569 as an indirect representative of the *E. oleifera* species. In the latter case, most mapped molecular markers were segregated from the interspecific parent SA65T, inherited from *E. oleifera* or (by contrast) *E. guineensis* grandparent genomes but monomorphic in the *E. guineensis* parent PO3228D [27]. A main cause of this monomorphism is the high homozygosity of PO3228D, a descendant by selfing of the Deli DA115D genitor, itself descending from only 4 ancestral palms of the Deli origin [48]. Moreover, Montoya *et al.* [27] showed that the detected QTLs for fatty acid composition in SA569 were only detected in the interspecific hybrid parent (SA65T), i.e. from both *Elaeis* grand-parent genomes while no statistical effect of any *E. guineensis* allele was evidenced in the *E. guineensis* parent PO3228D.

Comparative genome mapping across species or with other genomes has been used primarily to demonstrate events of synteny or, conversely, propensity for chromosome rearrangement and it has provided valuable insights into the evolution of genomes. Our comparison of intra- and interspecific *Elaeis* genetic maps showed a high degree of marker locus co-linearity. Consequently, a common set of highly transferable SSRs, with known genome positions, is available to search common or specific QTL regions in both *Elaeis* genomes. These types of results are frequently reported, for example, in the genus *Rubus* of sub-family Rosoideae [49], in the genus *Vigna* [50], and in *Eucalyptus* species [51].

The comparison of genetic maps of cowpea (*Vigna unguiculata* L. Walp) at the subspecies level (*Vigna unguiculata* ssp. *sesquipedalis*) or in broader species such as *Lotus japonica* and soybean had revealed differences between taxa but highlighted high conservation zones and the syntenic relationships between related crop legume species or subspecies [52]. A comparative genetic and QTL mapping experiment between white oaks (*Quercus robur L*. and *Q. petraea L*.) showed a significant number of co-locations for QTLs controlling the timing of bud burst. The differences between these species are based in the influence of environmental factors or the phenotypic plasticity inherent in these species [53].

The comparison of QTLs throughout the genetic maps of a single genus, in this case *Elaeis*, provides increased information on gene contributions to the phenotypic variation in fatty acid proportions in various genetic populations. Common genomic regions across populations, involving QTLs of interest, facilitate positional cloning and marker-assisted selection of agronomic genes. For example, this type of strategy has been used successfully between inter- (*Sorghum bicolor* L. Moench or *S. propinquum*) or intraspecific (*S. bicolor*) sorghum populations that showed a high degree of marker collinearity and correspondence for QTL regions associated with different traits [54].

QTL regions common to both *Elaeis* genomes are in accordance with the alignment of QTL maps in other, related species. However, these cases represented only two out of 15 QTL regions. This situation is similar to the results of Chen *et al.* [55] in tomato species, where, apart from common genomic positions, 75% and 85% of QTLs were species-specific for fruit weight and total soluble solid content, respectively.

Some QTLs in this analysis were surely missed or falsely identified due to the limited size of our mapping populations [56]. The QTL power detection in a cross is the same for common or specific QTLs at an equivalent QTL polymorphism. The apparent higher frequency of specific QTLs can be explained by parent homozygosity at QTLs/genes (therefore undetectable) in regions for one but not the other cross. Phenotypic variations might have been insufficient to identify some common QTLs, for instance in the intraspecific cross.

In fact, QTLs of different genetic types were revealed. On one hand, the oil palm cross identified only intraspecific *E. guineensis* QTLs. Through the heterozygous mapping parents, these QTLs are responsible for intra-phenotypic variations of the *E. guineensis* populations La Mé and Deli. On the other hand, the interspecific pseudo-backcross had two different types of QTLs: 1) purely intraspecific *E. oleifera* QTLs and 2) interspecific QTLs. The first type contributes to intra-phenotypic variations of the *E. oleifera* population. It can share (or not) common genomic regions with *E. guineensis* QTLs. The second type is responsible for between-species phenotypic differences. It corresponds to homozygous QTLs (fixed genes) in each species, with species-specific alleles responsible for between-species phenotypic differences. Such QTLs, undetectable in pure *E. guineensis* or *E. oleifera* mapping populations, likely make up the majority of QTLs detected in the backcross. Complementary mapping populations of *E. oleifera* and respective QTL map for fatty acid composition would enable us to compare and further validate the genomic regions associated with variations within each species, and help in exploring further if the genetic architecture of palm oil fatty acid composition is the same or different between the cultivated oil palm and its wild relative *E. oleifera*.

Two independent QTL regions, one on the *E. guineensis* cross (on LG 9) and the other on the *E. oleifera*-derived cross (on LG 6), can be considered most important because they each harbor QTLs for several traits with strong effects. This pattern could indicate that the phenotypic variability of the palm oil fatty acid composition is under the control of different regions of the genomes of these two species. However, our results are not sufficient to support this hypothesis and more work and analyses of complementary experiments should be undertaken before generalizing such hypothesis to the whole *E. guineensis* and *E. oleifera* species.

Furthermore, the colocalization of QTLs suggested either pleiotropic effects or linkage. This finding indicates that in these genomic regions, there is either a single segregating locus affecting the biosynthesis of several fatty acids pleiotropically or clusters of linked QTLs independently affecting the biosynthesis of the different fatty acids. Fine-mapping of these QTL regions and the analysis of future sequence data from oil palm will help to determine whether linkage or pleiotropy is responsible for this colocalization. In *E. guineensis*, the colocalization of major QTLs associated with C14:0, C16:0, C18:0, and C18:1 was in agreement with the phenotypic correlations we observed between those four fatty acids. These results are similar to those reported, for example, in jatropha [57] and oat [58]. In parallel, the detection of common QTL regions in LG 4 and LG 15 tends to confirm that these zones could be involved in the same genetic determinism for a portion of the variations regardless of the *Elaeis* species.

The genetic value of oil palm individuals in terms of the fatty acid composition of palm oil can be estimated easily (based on measurements on the first bunches produced by the individuals). Therefore, the main interest in molecular markers associated with fatty acid composition does not lie in marker-assisted selection but, rather, in the possibility of optimizing crosses between selected individuals to accumulate favorable genes. For instance, this process has been successfully implemented to increase the content of oleic acid in the peanut [59]. From an operational point of view, our study can be considered a first step toward achieving this goal in the oil palm, which will require more accurate estimates of QTL positions and estimates of their effects and, possibly, a QTL detection study extended to QTLs with smaller effects. QTLs associated with different fatty acids (through either linkage or pleiotropy) that colocalize in the same genomic regions will be difficult to use if their effects are opposite for saturated and unsaturated fatty acids. Obviously, a QTL associated with a single fatty acid will be easier to use. When QTLs colocalize in the same genomic regions for the two species for a given fatty acid, markers specific to the favorable species will have to be developed to follow the favorable alleles and accumulate them in *E. guineensis*.

We chose which QTLs to compare based on trials under different environmental conditions. Therefore, these QTLs might be biased by possible environmental effects on the fatty acid composition. However, the high heritability of fatty acid proportions and the location of both experiments in similar agro-climatic conditions suggest that the QTLs are accurate and comparable between sites. A future genotype x environment experiment should test this assertion.

We have no basis on which to formulate hypotheses on the genes underlying the *E. guineensis* QTLs. The few genes of palm oil biosynthesis mapped by Montoya *et al.* [27] in SA569 were outside these QTL regions. These QTL genes should be found by other methods, preferably based on the search, sequencing, and mapping of genes involved in *Elaeis* palm oil biosynthesis as well as gene expression studies of the pulp of developing fruits in various *Elaeis* genetic materials. These experiments will be a next step of our research.

Based on the phenotypic variability existing between *E. guineensis* and *E. oleifera* in the profile of fatty acids of their respective oils, we hypothesized that the genetic architecture of this trait would differ between these two species in terms of the number and position of QTLs and in the phenotypic variance they explain. In the studied *E. guineensis* intraspecific cross and interspecific pseudo-backcross, dense microsatellite linkage maps, with a high number of common and collinear SSR marker loci, allowed us to identify and compare QTLs. The results of the QTL detection undertaken in *E. guineensis* were interpreted in comparison with QTLs for the same traits in the interspecific pseudo-backcross. Evidence is a difference in terms of number and position of QTL between our intraspecific and interspecific mapping populations, which might be only due to differences in terms of QTL polymorphism between the studied genetic materials, not to presence or absence of coding sequences underlying QTL regions in the two *Elaeis* genomes. The whole genome sequences in oil palm and *E. oleifera* recently published by Singh *et al.* [61] will greatly help in solving at this level the genetic determinism of the palm oil fatty acid composition.

**Figure 3:**
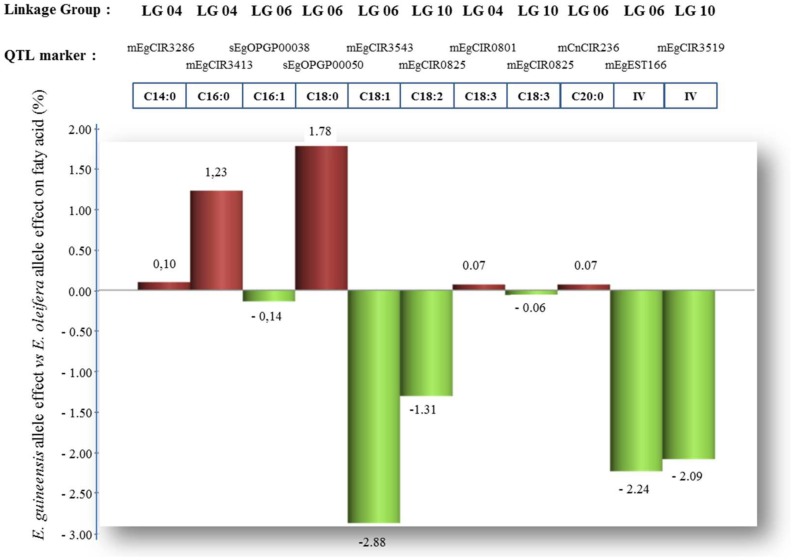
Effects of *E. guineensis versus E. oleifera* QTL alleles on the palm oil fatty acid composition, estimated by Montoya et al. [27] from the interspecific pseudo-backross SA569. Note: the QTL marker loci were used to perform an ANOVA test (type III, post hoc test of Tukey at α = 0.05) to estimate the mean effects of the parent QTL marker alleles on the mean of each phenotypic trait. For the hybrid parent SA65T, the species origin of the QTL marker alleles were identified, and the allelic effects at the QTL were therefore estimated by contrast of *E. oleifera* (grand-parent SA49D) against *E. guineensis* (grand-parent LM2466P).

Having said, we must consider also different *E. guineensis versus E. oleifera* QTL allele effects on the palm oil fatty acid composition (Figure 3), from QTL results of Montoya *et al.* [27] in the interspecific backcross SA569, in view to explore differences or similarities in gene expression between the two *Elaeis* genomes. Indeed, “the effects associated to the *E. guineensis* QTL marker alleles were positive for the proportions of saturated fatty acids C14:0, C16:0, C18:0, and C20:0. In parallel, they were negative for the percentage of the unsaturated fatty acids C16:1, C18:1, C18:2, and C18:3 and for the iodine value IV. Only for C18:3, the *E. guineensis* allele of the QTL locus mEgCIR0801 presented a negative effect.” This fact was in good coherence with both the knowledge of the oil biosynthesis pathway in plants and with the individual correlations estimated between the fatty acid proportions in the palm oil. Therefore, apart the QTL positions, the *E. guineensis* or *E. oleifera* species origin of the QTL alleles and their associated effects on the palm oil fatty acid composition will be characterized on the whole *Elaeis* genus by combining genetics and gene expression studies.

## Supporting Information

Figure S1 Pedigree of the intraspecific LM2T x DA10D and SA5569 crosses with examples of traceable *E. guineensis* or *E. oleifera* SSR segregating alleles. The cross LM2T x DA10D was considered a representative of the species *E. guineensis* and the interspecific pseudo-backcross SA569 as an indirect representative of the species *E. oleifera*.
(TIFF)

Figure S2 Histograms of the palm oil fatty acid proportions in the cross LM2T x DA10D.
(TIFF)

Table S1 Summary statistics of phenotypic traits in LM2T x DA10D (n = 71).
(PDF)

Table S2 Pearson's correlations between phenotypic traits and palm oil composition traits in LM2T x DA10D (n = 71).
(PDF)
