# Correction: Genetic Architecture of Palm Oil Fatty Acid Composition in Cultivated Oil Palm (*Elaeis guineensis* Jacq.) Compared to Its Wild Relative *E. oleifera* (H.B.K) Cortés

**DOI:** 10.1371/journal.pone.0101628

**Published:** 2014-06-24

**Authors:** The PLOS ONE Staff

Figure 2 is missing parts B and C. The authors have provided a corrected version below.

**Figure 2:**
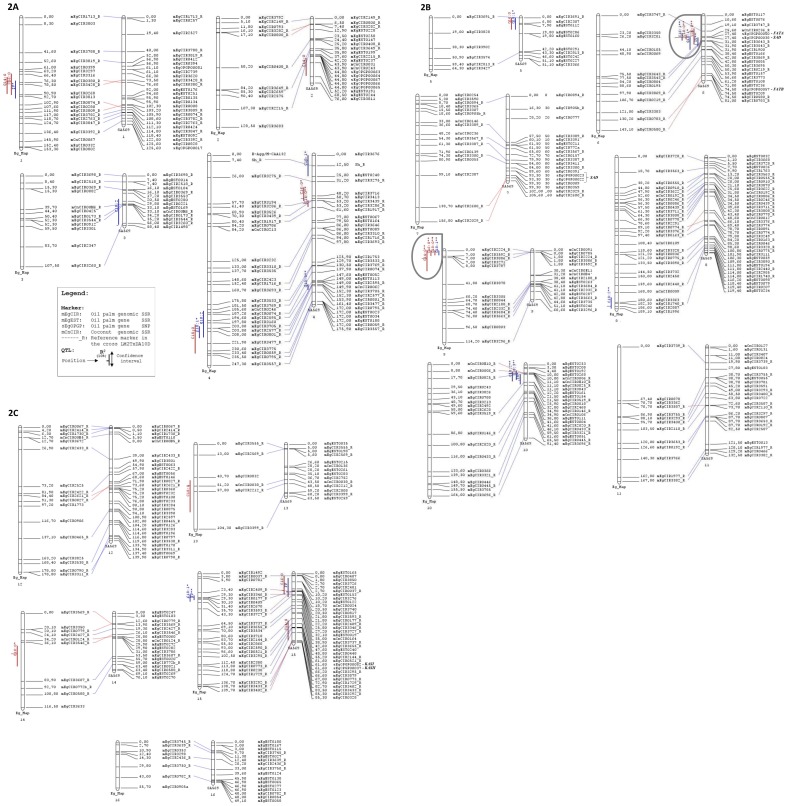
Sixteen QTLs of palm oil fatty acid proportions and iodine value identified in the *E. guineensis* cross LM2T x DA10D, located on the consensus linkage map in oil palm (Eg_Map) of Billotte *et al.* [36] and compared to the QTL map for same traits published by Montoya *et al.* [27] in the interspecific *Elaeis* pseudo-backcross SA569. Note: Each microsatellite linkage map has 16 linkage groups corresponding to the 16 homologous pairs of chromosomes of the *Elaeis* genome. The *E. guineensis* Eg_Map (253 loci) is sharing 156 marker loci in common and good co-linearity with the linkage map of the pseudo-backcross SA569 (362 loci). The QTLs were identified by the Kruskal-Wallis, IM and MQM methods. One star (*) or two stars (**): QTL detected by the MQM method at the genome-wide α threshold value of 5% or 1% respectively. No star: putative QTL as only detected by the Kruskall-Wallis test at p<0.005. The names and the positions (cM) of the markers are given on the right side of the linkage groups. mEgCIRxxxx and mEgESTxxxx: *E. guineensis* SSR loci. sEgOPGPxxxx: *E. guineensis* gene SNP loci. mCnCIRxxxx: *Cocos nucifera* SSR loci. Marker loci common to both maps are indicated by an extension “_R”. The names, positions and confidence regions of the QTLs are given on the left side of the linkage groups. In red: are figured the QTLs of saturated fatty acid proportion; in blue: the QTLs of unsaturated fatty acid proportion and of iodine value.
