# Supplementary figures and images for: Genetic Architecture of Palm Oil Fatty Acid Composition in Cultivated Oil Palm (Elaeis guineensis Jacq.) Compared to Its Wild Relative E. oleifera (H.B.K) Cortés
Source: PLoS One. 2014 May 9;9(5):e95412. doi: 10.1371/journal.pone.0095412 (PMC4015976; doi:10.1371/journal.pone.0095412)

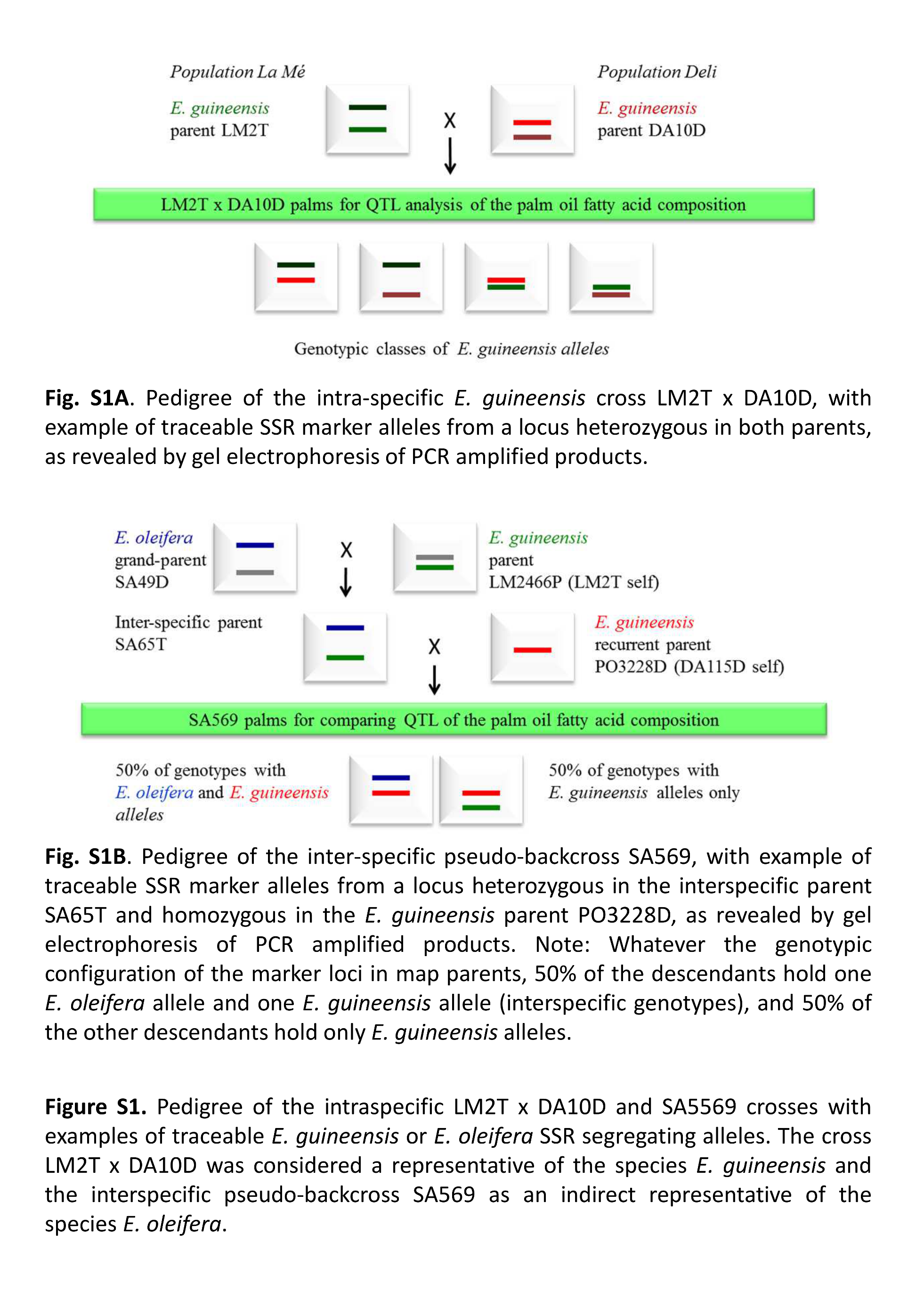

Supplement: Figure S1 — Pedigree of the intraspecific LM2T x DA10D and SA5569 crosses with examples of traceable E. guineensis or E. oleifera SSR segregating alleles. The cross LM2T x DA10D was considered a representative of the species E. guineensis and the interspecific pseudo-backcross SA569 as an indirect representative of the species E. oleifera. (TIFF) [file pone.0095412.s001.tif]

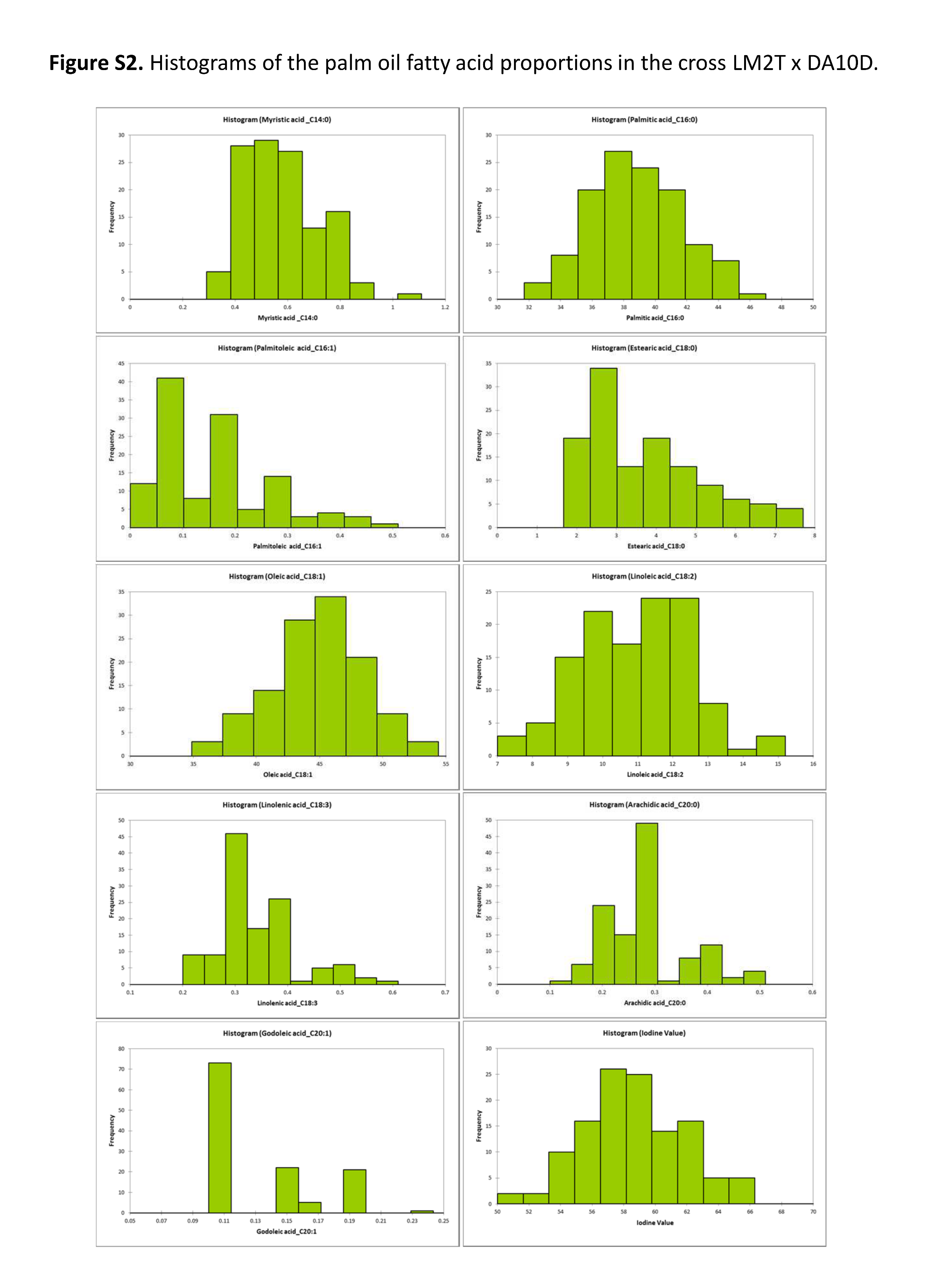

Supplement: Figure S2 — Histograms of the palm oil fatty acid proportions in the cross LM2T x DA10D. (TIFF) [file pone.0095412.s002.tif]
