# Supplementary material for: Genetic Architecture of Palm Oil Fatty Acid Composition in Cultivated Oil Palm (Elaeis guineensis Jacq.) Compared to Its Wild Relative E. oleifera (H.B.K) Cortés
Source: PLoS One. 2014 May 9;9(5):e95412. doi: 10.1371/journal.pone.0095412 (PMC4015976; doi:10.1371/journal.pone.0095412)
Supplement: Table S1 — Summary statistics of phenotypic traits in LM2T x DA10D (n = 71). (PDF) [file pone.0095412.s003.pdf]

Table S1: Summary statistics of phenotypic traits in LM2T x DA10D (n=71).

No.	Phenotypic Trait	Acronym	Estimation basis	Observations without missing data	Minimum	Maximum	Mean	Standard deviation
	Production:		***Systematic***					
1	Fruit variety	type	Morphology observation of the fruit section	116	-	-	-	-
2	Average bunch number/palm/year at 3-5 years	Bn3_5	individual observation at each harvest	71	22.3	35.1	30.0	2.5
3	Average bunch weigth at 3-5 years (kg)	Bwt3_5	calculation (Bwt3_5 = FFB3_5/Bn3_5)	71	3.7	6.2	4.7	0.5
4	Fresh Fruit Bunch yield/palm/year at 3-5 years (kg/palm/year)	FFB3_5	measure at each harvest	71	107.0	183.7	139.2	15.9
5	Palm oil yield/palm/year at 3-5 years (ton/ha/year)	PO3_5	calculation (135 productive palms/ha x FFB3_5 x IER)	71	3.3	6.0	4.3	0.6
6	Average bunch number/palm/year at 6-9 years	Bn6_9	individual observation at each harvest	71	17.2	25.2	21.8	2.0
7	Average bunch weigth at 6-9 years (kg)	Bwt6_9	calculation (Bwt6_9 = FFB6_9/Bn6_9)	71	9.1	15.1	11.3	1.1
8	Fresh Fruit Bunch yield/palm/year at 6-9 years (kg/palm/year)	FFB6_9	measure at each harvest	71	157.7	300.8	242.4	28.2
9	Palm oil yield/palm/year at 6-9 years (ton/ha/year)	PO6_9	calculatation (135 productive palms/ha x FFB6_9 x IER)	71	4.8	10.2	7.5	1.0
	Bunch components		***Measure (from 8 bunches analysed per palm)***					
10	Average weigth of the analysed bunch (kg)	aBwt	measure	71	10.4	20.0	14.4	2.0
11	Average number of spickelets per bunch	Spikelets	measure	71	98.5	255.8	146.3	25.2
12	Average number of fruits per bunch	Fn	calculatation (Fn = aBwt/Fwt)	71	819.2	2049.8	1255.8	220.9
13	Average weigth of the fruit (g)	Fwt	estimation on fruits sample	71	5.8	10.0	7.6	1.0
14	Fruit to bunch ratio (%)	%FB	measure	71	57.9	69.2	63.8	2.3
15	Pulp to fruit ratio (%)	%PF	estimation on fruits sample	71	70.2	86.5	77.6	3.1
16	Palm oil to pulp ratio (%)	%POP	estimation on fruits sample	71	47.6	59.0	54.7	2.5
17	Palm oil industrial extraction rate (%)	IER	calculation (IER = 0.855 x %FB x %PF x %POP/10000)	71	17.6	27.4	23.0	2.1
18	Kernel to fruit ratio (%)	%KF	estimation on fruits sample	71	6.1	13.3	9.5	1.4
	Vegetative growth:		***Systematic***					
19	Stem height (m)	Ht	1 measure at 15 years old, at the level of the Leaf 33	71	541.6	821.7	654.5	62.4
20	Average number of leaves per crown	L17_L	6 measures at 15 years old	71	553.5	716.5	634.0	34.0
21	Average length of the leaf L17 (cm)	P_W	6 measures at 15 years old	71	7.6	11.0	9.1	0.7
22	Petiole average width of the leaf L17 (cm)	P_T	6 measures at 15 years old	71	3.6	5.5	4.7	0.4
23	Petiole average thickness of the leaf L17 (cm)	Leaf_n	6 measures at 15 years old	71	22.9	44.4	34.0	4.2
24	Average number of leaflets per leaf L17	Lt_n	6 measures at 15 years old	71	154.3	196.2	170.9	8.7
25	Leaflet average length of the leaf L17 (cm)	Lt_L	6 measures at 15 years old	71	90.9	121.5	104.5	6.6
26	Leaflet average width of the leaf L17 (cm)	Lt_W	6 measures at 15 years old	71	5.4	7.5	6.4	0.5
	**Palm Oil quality:**		***Measure (from 2 bunches analysed per palm)***					
27	% Myristic acid (C14:0)	Myristic acid_C14:0		71	0.3	1.0	0.5	0.2
28	% Palmitic acid (C16:0)	Palmitic acid_C16:0	Fatty acids extracted by a standard procedure	71	35.2	49.7	40.9	3.0
29	% Palmitoleic acid (C16:1)	Palmitoleic acid_C16:1	and converted into fatty acid methyl esters.	71	0.1	0.2	0.1	0.0
30	% Stearic acid (C18:0)	Stearic acid_C18:0	(FAMEs) and FAMEanalyzed by gas chromatography	71	3.8	7.0	5.1	0.8
31	% Oleic acid (C18:1)	Oleic acid_C18:1	(GC) according to the AFNOR method NF T60-233/1977	71	35.4	48.9	43.3	2.8
32	% Linoleic acid (C18:2)	Linoleic acid_C18:2	(AFNOR, 1977) using Tchobo et al. (2007) protocol	71	6.9	12.1	9.3	0.9
33	% Linolenic acid (C18:3)	Linolenic acid_C18:3	with minor modifications.	71	0.2	0.4	0.2	0.1
34	% Arachidic acid (C20:0)	Arachidic acid_C20:0		71	0.2	0.5	0.4	0.1
35	% Godoleic acid (C20:1).	Gadoleic acid_C20:1		71	0.1	0.4	0.1	0.0
36	% Saturated	Saturated	calculation	71	41.7	54.5	46.9	2.6
37	% Monounsaturated	Monounsaturated	calculation	71	27.2	49.1	43.3	3.4
38	% Polyunsaturated	Polyunsaturated	calculation	71	7.1	26.1	9.8	2.2
39	Ratio 16:0/18:1	16:0/18:1	calculation	71	0.7	1.4	1.0	0.1
40	Ratio 18:1/16:0	18:1/16:0	calculation	71	0.7	1.4	1.1	0.1
41	Iodine value (proportion of unsaturated fatty acids)	IV	Wijs method described in the ISO 3961:2009 standard	71	49.4	58.9	54.7	1.9
