# Supplementary material for: Genetic Architecture of Palm Oil Fatty Acid Composition in Cultivated Oil Palm (Elaeis guineensis Jacq.) Compared to Its Wild Relative E. oleifera (H.B.K) Cortés
Source: PLoS One. 2014 May 9;9(5):e95412. doi: 10.1371/journal.pone.0095412 (PMC4015976; doi:10.1371/journal.pone.0095412)
Supplement: Table S2 — Pearson's correlations between phenotypic traits and palm oil composition traits in LM2T x DA10D (n = 71). (PDF) [file pone.0095412.s004.pdf]

Table S2: Pearson's correlations between phenotypic traits and palm oil composition traits in LM2T x DA10D (n=71).

Correlation matrix (Pearson) - *Part 1:*

	Variables	Production								Bunch Components								
		Bn3_5	Bwt3_5	FFB3_5	PO3_5	Bn6_9	Bwt6_9	FFB6_9	PO6_9	aBwt	Spikelets	Fn	Fwt	%FB	%PF	%POP	IER	%KF
Production	Bn3_5	**1**	-0.108	**0.488**	**0.314**	**0.488**	-0.135	**0.444**	**0.278**	-0.110	0.005	-0.080	-0.056	-0.054	-0.081	-0.112	-0.066	0.057
	Bwt3_5	-0.108	**1**	**0.775**	**0.607**	**-0.302**	**0.607**	**0.266**	**0.238**	**0.395**	**0.237**	0.129	**0.300**	0.106	0.031	-0.016	0.029	0.000
	FFB3_5	**0.488**	**0.775**	**1**	**0.773**	0.118	**0.373**	**0.526**	**0.428**	**0.282**	0.210	0.051	**0.261**	0.098	0.014	-0.035	0.047	0.020
	PO3_5	**0.314**	**0.607**	**0.773**	**1**	0.084	**0.304**	**0.405**	**0.736**	**0.398**	**0.265**	0.176	**0.302**	**0.322**	**0.358**	**0.414**	**0.654**	**-0.238**
	Bn6_9	**0.488**	**-0.302**	0.118	0.084	**1**	**-0.478**	**0.609**	**0.450**	**-0.497**	**-0.284**	**-0.392**	-0.006	0.068	0.061	0.047	0.021	-0.029
	Bwt6_9	-0.135	**0.607**	**0.373**	**0.304**	**-0.478**	**1**	**0.362**	**0.304**	**0.525**	**0.378**	**0.311**	0.195	0.006	-0.036	0.013	0.006	0.026
	FFB6_9	**0.444**	**0.266**	**0.526**	**0.405**	**0.609**	**0.362**	**1**	**0.760**	-0.066	0.051	-0.183	0.219	0.029	0.034	0.069	0.016	0.002
	PO6_9	**0.278**	**0.238**	**0.428**	**0.736**	**0.450**	**0.304**	**0.760**	**1**	0.143	0.143	0.006	**0.267**	**0.295**	**0.403**	**0.502**	**0.650**	**-0.271**
Bunch Components	aBwt	-0.110	**0.395**	**0.282**	**0.398**	**-0.497**	**0.525**	-0.066	0.143	**1**	**0.678**	**0.749**	**0.275**	**0.406**	0.079	0.029	**0.286**	-0.100
	Spikelets	0.005	**0.237**	0.210	**0.265**	**-0.284**	**0.378**	0.051	0.143	**0.678**	**1**	**0.618**	0.115	**0.271**	0.029	-0.079	0.149	-0.147
	Fn	-0.080	0.129	0.051	0.176	**-0.392**	**0.311**	-0.183	0.006	**0.749**	**0.618**	**1**	**-0.358**	**0.482**	0.045	-0.047	**0.242**	-0.143
	Fwt	-0.056	**0.300**	**0.261**	**0.302**	-0.006	0.195	0.219	**0.267**	**0.275**	0.115	**-0.358**	**1**	0.064	0.065	0.106	0.117	0.022
	%FB	-0.054	0.106	0.098	**0.322**	0.068	0.006	0.029	**0.295**	**0.406**	**0.271**	**0.482**	0.064	**1**	0.052	-0.017	**0.430**	-0.044
	%PF	-0.081	0.031	0.014	**0.358**	0.061	-0.036	0.034	**0.403**	0.079	0.029	0.045	0.065	0.052	**1**	**0.340**	**0.572**	**-0.773**
	%POP	-0.112	-0.016	-0.035	**0.414**	0.047	0.013	0.069	**0.502**	0.029	-0.079	-0.047	0.106	-0.017	**0.340**	**1**	**0.718**	-0.213
	IER	-0.066	0.029	0.047	**0.654**	0.021	0.006	0.016	**0.650**	**0.286**	0.149	**0.242**	0.117	**0.430**	**0.572**	**0.718**	**1**	**-0.422**
	%KF	0.057	0.000	0.020	**-0.238**	-0.029	0.026	0.002	**-0.271**	-0.100	-0.147	-0.143	0.022	-0.044	**-0.773**	-0.213	**-0.422**	**1**
Vegetative growth	Ht	0.109	0.014	0.062	0.069	0.228	0.098	**0.355**	**0.294**	0.113	0.169	0.047	0.126	0.126	0.144	-0.091	0.035	**-0.281**
	Leaf_n	0.080	**0.298**	**0.311**	0.218	-0.188	**0.255**	0.047	0.011	**0.406**	**0.363**	**0.316**	0.057	0.068	-0.171	-0.082	-0.041	0.073
	L17_L	-0.091	-0.042	-0.066	-0.044	-0.019	-0.088	0.002	0.003	-0.176	-0.101	**-0.246**	0.102	-0.194	0.183	0.104	-0.005	0.024
	P_W	-0.123	0.193	0.059	0.037	-0.105	**0.345**	**0.234**	0.162	0.228	0.121	0.026	**0.297**	0.075	-0.104	0.053	-0.043	0.167
	P_T	-0.193	**0.278**	0.099	0.176	-0.123	**0.300**	0.210	**0.253**	0.188	0.178	0.036	0.219	0.019	**0.262**	0.173	0.125	-0.135
	Lt_n	0.135	-0.038	0.080	-0.002	0.194	-0.128	0.134	0.021	-0.216	-0.151	**-0.429**	**0.237**	**-0.316**	0.030	0.046	-0.135	0.040
	Lt_L	**-0.250**	0.126	-0.027	0.140	-0.083	0.203	0.090	**0.249**	**0.269**	0.015	0.115	0.205	0.182	0.073	0.209	**0.246**	0.097
	Lt_W	0.008	0.172	0.124	0.082	0.075	**0.338**	**0.403**	**0.294**	-0.091	-0.073	**-0.287**	**0.298**	-0.129	-0.079	0.014	-0.070	-0.029
Palm Oil Composition	C14:0	0.179	-0.074	-0.021	-0.075	-0.127	0.174	0.048	-0.032	0.048	-0.008	0.005	-0.022	0.024	-0.118	-0.149	-0.096	0.009
	C16:0	**0.277**	-0.003	0.088	-0.035	-0.036	0.077	0.085	-0.035	-0.093	-0.067	-0.199	0.101	-0.071	-0.086	-0.131	-0.131	-0.012
	C16:1	0.024	-0.043	-0.025	-0.130	**-0.262**	0.091	-0.133	-0.222	**0.244**	**0.288**	0.221	-0.042	0.077	-0.188	**-0.306**	-0.171	0.024
	C18:0	**-0.249**	-0.046	-0.158	-0.088	-0.035	-0.171	-0.189	-0.104	-0.063	-0.055	0.048	-0.119	-0.044	0.030	0.121	0.035	0.062
	C18:1	**-0.243**	0.018	-0.071	0.051	0.023	-0.036	-0.065	0.050	0.076	0.026	0.192	-0.120	0.148	0.101	0.078	0.131	0.016
	C18:2	0.002	-0.024	0.030	0.018	0.133	-0.049	0.076	0.062	0.090	0.172	0.033	0.104	-0.143	0.006	0.111	0.024	-0.094
	C18:3	0.149	0.029	0.112	0.114	0.060	0.187	**0.291**	**0.249**	0.074	-0.077	-0.037	0.148	0.050	-0.076	0.056	0.052	0.082
	C20:0	**-0.263**	0.165	-0.005	-0.045	0.023	0.031	0.019	-0.009	-0.087	-0.203	-0.166	0.106	-0.147	0.029	0.177	-0.077	-0.004
	C20:1	-0.148	0.054	-0.024	0.035	-0.196	0.051	-0.149	-0.080	0.134	0.050	0.113	0.010	-0.102	-0.106	0.117	0.053	0.054
	IV	-0.223	-0.039	-0.053	0.031	0.140	-0.154	-0.051	0.036	0.078	0.054	0.169	-0.085	0.027	0.085	0.133	0.110	-0.010

Values in bold are different from 0 with a significance level alpha=0,05

Correlation matrix (Pearson) - *Part 2:*

	Variables	Vegetative growth								Palm Oil Composition									
		Ht	L17_L	P_W	P_T	Leaf_n	Lt_n	Lt_L	Lt_W	C14:0	C16:0	C16:1	C18:0	C18:1	C18:2	C18:3	C20:0	C20:1	IV
Production	Bn3_5	0.109	0.080	-0.091	-0.123	-0.193	0.135	**-0.250**	0.008	0.179	**0.277**	0.024	**-0.249**	**-0.243**	0.002	0.149	**-0.263**	-0.148	-0.223
	Bwt3_5	0.014	**0.298**	-0.042	0.193	**0.278**	-0.038	0.126	0.172	-0.074	-0.003	-0.043	-0.046	0.018	-0.024	0.029	0.165	0.054	-0.039
	FFB3_5	0.062	**0.311**	-0.066	0.059	0.099	0.080	-0.027	0.124	-0.021	0.088	-0.025	-0.158	-0.071	0.030	0.112	-0.005	-0.024	-0.053
	PO3_5	0.069	0.218	-0.044	0.037	0.176	-0.002	0.140	0.082	-0.075	-0.035	-0.130	-0.088	0.051	0.018	0.114	-0.045	0.035	0.031
	Bn6_9	0.228	-0.188	-0.019	-0.105	-0.123	0.194	-0.083	0.075	-0.127	-0.036	**-0.262**	-0.035	0.023	0.133	0.060	0.023	-0.196	0.140
	Bwt6_9	0.098	**0.255**	-0.088	**0.345**	**0.300**	-0.128	0.203	**0.338**	0.174	0.077	0.091	-0.171	-0.036	-0.049	0.187	0.031	0.051	-0.154
	FFB6_9	**0.355**	0.047	0.002	**0.234**	0.210	0.134	0.090	**0.403**	0.048	0.085	-0.133	-0.189	-0.065	0.076	**0.291**	0.019	-0.149	-0.051
	PO6_9	**0.294**	0.011	0.003	0.162	**0.253**	0.021	**0.249**	**0.294**	-0.032	-0.035	-0.222	-0.104	0.050	0.062	**0.249**	-0.009	-0.080	0.036
Bunch Components	aBwt	0.113	**0.406**	-0.176	0.228	0.188	-0.216	**0.269**	-0.091	0.048	-0.093	**0.244**	-0.063	0.076	0.090	0.074	-0.087	0.134	0.078
	Spikelets	0.169	**0.363**	-0.101	0.121	0.178	-0.151	0.015	-0.073	-0.008	-0.067	**0.288**	-0.055	0.026	0.172	-0.077	-0.203	0.050	0.054
	Fn	0.047	**0.316**	**-0.246**	0.026	0.036	**-0.429**	0.115	**-0.287**	0.005	-0.199	0.221	0.048	0.192	0.033	-0.037	-0.166	0.113	0.169
	Fwt	0.126	0.057	0.102	**0.297**	0.219	**0.237**	0.205	**0.298**	-0.022	0.101	-0.042	-0.119	-0.120	0.104	0.148	0.106	0.010	-0.085
	%FB	0.126	0.068	-0.194	0.075	0.019	**-0.316**	0.182	-0.129	0.024	-0.071	0.077	-0.044	0.148	-0.143	0.050	-0.147	-0.102	0.027
	%PF	0.144	-0.171	0.183	-0.104	**0.262**	0.030	0.073	-0.079	-0.118	-0.086	-0.188	0.030	0.101	0.006	-0.076	0.029	-0.106	0.085
	%POP	-0.091	-0.082	0.104	0.053	0.173	0.046	0.209	0.014	-0.149	-0.131	**-0.306**	0.121	0.078	0.111	0.056	0.177	0.117	0.133
	IER	0.035	-0.041	-0.005	-0.043	0.125	-0.135	**0.246**	-0.070	-0.096	-0.131	-0.171	0.035	0.131	0.024	0.052	-0.077	0.053	0.110
	%KF	**-0.281**	0.073	0.024	0.167	-0.135	0.040	0.097	-0.029	0.009	-0.012	0.024	0.062	0.016	-0.094	0.082	-0.004	0.054	-0.010
Vegetative growth	Ht	**1**	0.175	**-0.316**	0.148	0.232	-0.059	-0.011	**0.372**	0.086	-0.011	-0.036	0.007	0.007	-0.005	-0.007	0.130	0.048	-0.023
	Leaf_n	0.175	**1**	-0.180	0.085	-0.005	-0.056	-0.036	-0.005	0.029	-0.038	0.101	0.011	-0.025	0.143	0.101	-0.073	0.106	0.022
	L17_L	**-0.316**	-0.180	**1**	**0.262**	**0.400**	**0.345**	0.109	-0.006	-0.072	0.052	0.093	0.097	-0.086	0.024	0.126	-0.020	-0.024	0.019
	P_W	0.148	0.085	**0.262**	**1**	**0.625**	0.186	**0.254**	**0.340**	0.104	0.142	0.225	-0.060	-0.086	-0.169	**0.393**	-0.061	-0.045	-0.188
	P_T	0.232	-0.005	**0.400**	**0.625**	**1**	0.113	0.107	**0.276**	-0.186	-0.073	0.075	0.134	0.097	-0.105	0.115	0.002	-0.053	-0.022
	Lt_n	-0.059	-0.056	**0.345**	0.186	0.113	**1**	0.023	0.097	0.110	**0.259**	0.043	**-0.280**	**-0.241**	0.081	0.076	0.031	0.115	-0.103
	Lt_L	-0.011	-0.036	0.109	**0.254**	0.107	0.023	**1**	-0.039	-0.022	-0.122	-0.123	0.101	0.089	0.029	0.175	0.182	0.105	0.137
	Lt_W	**0.372**	-0.005	-0.006	**0.340**	**0.276**	0.097	-0.039	**1**	0.065	0.158	0.025	-0.146	-0.095	-0.143	**0.264**	0.097	0.002	-0.192
Palm Oil Composition	C14:0	0.086	0.029	-0.072	0.104	-0.186	0.110	-0.022	0.065	**1**	**0.733**	**0.391**	**-0.604**	**-0.727**	0.126	**0.381**	**-0.398**	-0.109	**-0.642**
	C16:0	-0.011	-0.038	0.052	0.142	-0.073	**0.259**	-0.122	0.158	**0.733**	**1**	**0.374**	**-0.773**	**-0.921**	0.058	0.205	**-0.429**	-0.138	**-0.858**
	C16:1	-0.036	0.101	0.093	0.225	0.075	0.043	-0.123	0.025	**0.391**	**0.374**	**1**	**-0.375**	**-0.379**	0.128	**0.336**	**-0.450**	0.020	**-0.247**
	C18:0	0.007	0.011	0.097	-0.060	0.134	**-0.280**	0.101	-0.146	**-0.604**	**-0.773**	**-0.375**	**1**	**0.598**	-0.045	-0.154	**0.496**	0.089	**0.553**
	C18:1	0.007	-0.025	-0.086	-0.086	0.097	**-0.241**	0.089	-0.095	**-0.727**	**-0.921**	**-0.379**	**0.598**	**1**	**-0.387**	**-0.254**	**0.340**	0.129	**0.737**
	C18:2	-0.005	0.143	0.024	-0.169	-0.105	0.081	0.029	-0.143	0.126	0.058	0.128	-0.045	**-0.387**	**1**	0.089	-0.014	-0.120	0.220
	C18:3	-0.007	0.101	0.126	**0.393**	0.115	0.076	0.175	**0.264**	**0.381**	0.205	**0.336**	-0.154	**-0.254**	0.089	**1**	-0.154	-0.067	-0.118
	C20:0	0.130	-0.073	-0.020	-0.061	0.002	0.031	0.182	0.097	**-0.398**	**-0.429**	**-0.450**	**0.496**	**0.340**	-0.014	-0.154	**1**	0.117	**0.376**
	C20:1	0.048	0.106	-0.024	-0.045	-0.053	0.115	0.105	0.002	-0.109	-0.138	0.020	0.089	0.129	-0.120	-0.067	0.117	**1**	0.109
	IV	-0.023	0.022	0.019	-0.188	-0.022	-0.103	0.137	-0.192	**-0.642**	**-0.858**	**-0.247**	**0.553**	**0.737**	0.220	-0.118	**0.376**	0.109	**1**

Values in bold are different from 0 with a significance level alpha=0,05
